# Leveraging Communication to Combat Antibiotic Resistance: A Longitudinal Test of a Video-Based Intervention to Improve Providers’ Stewardship Skills

**DOI:** 10.3390/antibiotics14121270

**Published:** 2025-12-15

**Authors:** Yanmengqian Zhou, Madeline Jupina, Elizabeth Gibbs, Bryan Mesquita, Erina L. Farrell

**Affiliations:** 1 Department of Communication, University at Buffalo, Buffalo, NY 14260, USA; 2 Department of Communication Arts & Sciences, Pennsylvania State University, University Park, PA 16802, USA; mmj5672@psu.edu (M.J.); bjm6826@psu.edu (B.M.); erinafarrell@psu.edu (E.L.F.); 3 Department of Psychology, Pennsylvania State University, University Park, PA 16802, USA; emg5623@psu.edu

**Keywords:** health communication, provider–patient communication, video-based intervention, longitudinal assessment, antibiotic stewardship, antibiotic resistance

## Abstract

**Background:** Antibiotic resistance is a pressing public health concern, exacerbated by the prescribing of antibiotics in primary care settings when they are not clinically indicated. Research shows that providers often feel pressured to prescribe antibiotics in response to patients’ expectations. To address this challenge, we developed a theory-informed video intervention to enhance antibiotic stewardship communication skills among current and future primary care providers, with emphasis on college health settings. The intervention consisted of five videos targeting core skills: explaining diagnosis and treatment, discussing antibiotic risks, advising on symptom management, offering patient support, and navigating visits with emerging adults. Program effectiveness was assessed immediately and three months post-intervention. **Methods:** Providers and medical students (*N* = 135) completed a three-wave study. At baseline, they reported demographics, perceived importance of the five skill sets, as well as self-efficacy, and completed closed- and open-ended skill assessment. Two weeks later, participants viewed the intervention videos, reassessed their skills, and rated their motivation to improve. Three months after video exposure, they completed the same skill assessments. **Results:** Participants showed improvements in all communication skills immediately post-intervention, except for supporting patients. While some skills showed sustained improvements at three months, the overall long-term effects were less pronounced. Perceived skill importance, baseline self-efficacy, and post-intervention motivation moderated these effects. **Conclusions:** A brief video-based intervention effectively enhanced current and future providers’ antibiotic stewardship communication skills. Sustaining these gains, however, requires ongoing reinforcement. Notably, the intervention was especially beneficial for those with low motivation and self-efficacy, key targets for such programs.

## 1. Introduction

Antibiotic resistance is a major public health concern, driven by inappropriate antibiotic use, particularly in outpatient settings [1]. According to the Centers for Disease Control and Prevention, there is a significant and growing threat from multiple antibiotic-resistant pathogens, including carbapenem-resistant *Enterobacterales*, carbapenem-resistant *Acinetobacter*, *Candida auris*, vancomycin-resistant *Enterococcus*, extended-spectrum beta-lactamase-producing *Enterobacterales*, and multidrug-resistant *Pseudomonas aeruginosa* [2]. Antibiotic resistance causes over 2.8 million infections and 35,000 deaths annually in the U.S. [3], with numbers expected to rise steadily if no action is taken. Public misconceptions and low risk perceptions contribute to the demand for antibiotics [4,5], creating pressure for providers to prescribe [6]. While patient-focused interventions are helpful [7], research suggests that empowering providers to delay or refuse prescriptions is more effective in promoting appropriate use [8].

As key gatekeepers, providers face significant challenges in managing patient expectations. Patients engage in various communication behaviors to pressure providers, such as suggesting diagnoses, referencing past prescriptions, and directly requesting antibiotics [9,10,11]. These behaviors not only create discomfort and defensiveness among providers but also lead to unnecessary prescriptions due to concerns about patient satisfaction and potential repercussions [10,11,12].

Evidence suggests that communication training may help providers navigate these pressures, supporting their efforts in promoting antibiotic stewardship while maintaining patient satisfaction [13,14]. Yet, systematic and scalable training programs that equip providers with these communication strategies remain limited. To address this gap, in this study, we developed and tested the longitudinal effectiveness of an intervention program, “Communicating for Antibiotic Stewardship,” that provides evidence-based training in provider communication skills to reduce patient expectations for antibiotics. The program was designed with a focus on primary care providers practicing at college health centers. This focus is driven by the high frequency of acute respiratory visits involved in student health practices, influenced by the living and hygiene conditions within the college student population [15,16]. However, much of the intervention content is relevant to other primary care contexts.

### 1.1. Communicating for Antibiotic Stewardship

Existing research highlights key communication strategies to reduce patient demands for antibiotics, such as diagnostic/treatment foreshadowing, explaining diagnoses and disease, discussing risks, offering follow-up plans, and engaging in patient-centered communication [10,11]. Initial evidence supports the effectiveness of training in these skills, highlighting the need for further development of practical interventions along these lines [13,14]. Guided by prior research and through an iterative consultation process with practitioners who were part of the antibiotic stewardship committee at our institution’s student health center, we developed theoretically grounded animated videos targeting five essential communication skills for primary healthcare providers practicing at college health centers.

#### 1.1.1. Explaining Diagnosis and Treatment

A key factor that predicts patients’ desire for antibiotics is their misconceptions about the drug and their illness [17]. A common misconception, for example, is that antibiotics can kill viruses. Patients with viral illness sometimes also mistakenly believe they have a bacterial infection. To reduce patient expectations, it is crucial for providers to logically present diagnostic and treatment information in ways that improve patients’ understanding of their illness, its appropriate treatment, and how they are connected. Informed by Toulmin’s model of argumentation [18], this video describes four communication strategies: *share reasoning for the diagnosis*, *address patient misconceptions*, *specify the diagnosis as viral*, and *explain how antibiotics relate to bacterial and viral illness*.

#### 1.1.2. Conveying the Risks of Antibiotics

Many individuals do not recognize the risks of antibiotics [4]. Theories and research on threat/fear appeals have consistently shown that presenting risk information can effectively enhance risk perceptions and change attitudes and behaviors [19]. Therefore, this video provides strategies for discussing the risks associated with antibiotics, particularly focusing on their *adverse effects* and the issue of *antibiotic resistance*, with an emphasis on depicting the severity and susceptibility and the role providers and patients can play in combating the threat [20]. In addition, since providers often face challenges in finding opportunities to discuss risks with patients [11], this video presents a strategy to help providers *find a space to talk about risks.*

#### 1.1.3. Advising on Symptom Management

Patients often seek antibiotics driven by the desire to alleviate their symptoms, and they can be satisfied when given alternative recommendations for managing their illness [21]. This underscores the importance for providers to offer effective symptom management advice to patients with viral illnesses. According to advice response theory (ART) [22], individuals are more likely to follow the advice when they view the advocated action as feasible, efficacious, and limitation-free. Guided by ART and research that applies ART in patient–provider communication [23], this video features five strategies: *describing symptom management as “real medicine,” emphasizing effectiveness and feasibility*, *addressing advice limitations and consequences*, *giving instructions for use*, and *helping patients follow through*.

#### 1.1.4. Supporting Patients

Patients who are not prescribed antibiotics may feel dissatisfied due to the perception that their providers are dismissive of their discomfort, lack compassion, and not taking their symptoms seriously [24]. Providers thus need to effectively convey support to their patients. Indeed, patient-centered care, which is characterized by empathy, partnership, and perspective-taking, is universally acknowledged as a key aspect of quality healthcare [25]. Research shows that perceived compassionate care from providers is associated with acceptance of non-antibiotic treatment [26]. Informed by models of patient-centered communication [27] and research on verbal-person centeredness [28], four strategies for supporting patients are presented in this video: *establishing a connection*, *drawing out the patient perspective*, *validating patient emotions and symptoms*, and *emphasizing the availability of follow-up care*.

#### 1.1.5. Navigating Medical Visits with Emerging Adults

Emerging adults, aged 18 to 27, constitute the primary patient population seen by our target audience. The theory of emerging adulthood considers this group who are at a transitioning period of life as distinct from individuals at other life stages. This age period is characterized by identity exploration, instability, self-focus, feeling in-between, and a sense of possibilities [29,30]. During this time, emerging adults begin to assert their autonomy and make independent life decisions [29], including health-related ones. However, as they take on more responsibility for their health, they may experience high levels of uncertainty due to inexperience and face conflicting health advice from various sources such as parents and peers. Building on the theory of emerging adulthood, this video outlines strategies for effectively communicating with this population, including *validating uncertainty, addressing competing sources of advice, and respecting patient autonomy*.

### 1.2. Animated Videos as an Educational Tool

The use of animated videos to present training materials is grounded in theory and research that highlight the value of this format in instructional settings. According to dual coding theory [31], individuals process information through interconnected verbal and imagery systems, and presenting information both verbally and visually facilitates information retrieval and enhances learning outcomes. The cognitive theory of multimedia learning [32] similarly posits that learners process information through dual channels–auditory and visual. It suggests that the simultaneous representation of words and pictures is more effective for learning than words alone. Indeed, a meta-analysis demonstrated that animated videos significantly enhance patient knowledge compared to standard methods such as written communication [33]. Provider-oriented interventions using animated videos have shown similar effectiveness [34]. Collectively, the evidence indicates that animated videos serve as an effective educational tool for enhancing healthcare providers’ skills in communicating antibiotic stewardship.

### 1.3. The Current Study

The primary objective of this study is to assess the effectiveness of our communication-focused intervention program. Specifically, we seek to evaluate both the immediate and longer-term (i.e., three months post-exposure) impacts of our training videos. Given the theoretical underpinning of these videos and the findings of prior research [13,35], we predict that there will be an immediate improvement in participants’ communication skills for discussing antibiotics with patients. However, there has been mixed evidence regarding the sustained effects of such interventions [36,37], we therefore propose a research question regarding the longer-term effects.

H1: Compared to their baseline levels, participants’ communication skills will demonstrate improvement immediately after watching the intervention videos.

RQ1: Will the effects of the intervention videos persist after two weeks?

The effectiveness of any intervention program often varies depending on individual factors [38]. Therefore, gaining a fuller understanding of the impacts of our intervention requires the examination of potential moderators. This not only allows us to identify specific conditions under which our intervention is most impactful but also facilitates future tailoring of intervention strategies to better meet the diverse needs of healthcare providers. Drawing on insights from previous theory and research [39,40,41], we focus on three potential moderators: perceived importance of the skills, baseline self-efficacy in executing the skills, and motivation to improve the skills. The following research question is proposed accordingly:

RQ2: Will the effects of the intervention video be moderated by participants’ (a) perceived skill importance, (b) baseline self-efficacy, and (c) motivation to improve?

## 2. Results

### 2.1. Immediate and Sustained Effects of the Intervention

Table 1 summarizes the fixed effects in models predicting the close- and open-ended communication skill assessments, both individually and as a whole. Time had a significant positive linear effect on participants’ overall communication skills measured with close-ended questions. This indicates that, consistent with H1, participants’ communication skills improved following the intervention, rather than showing no measurable change over time. Pairwise comparisons of the estimated marginal means (EMMs) with Tukey adjustment revealed significant improvement immediately after watching the videos (*M* = 28.72, *SE* = 0.11, *p* = 0.002) and at three months post-intervention (*M* = 28.67, *SE* = 0.10, *p* < 0.001) compared to baseline (*M* = 28.25, *SE* = 0.12). Testing changes in specific skill sets, time had a positive effect on explaining diagnosis and treatment and on navigating visits with emerging adults, indicating improvement in these targeted skills over time. Post hoc analyses demonstrated significant immediate (*M* = 5.84, *SE* = 0.03, *p* < 0.001) and long-term improvements (*M* = 5.84, *SE* = 0.03, *p* < 0.001) in explaining diagnosis and treatment compared to baseline (*M* = 5.60, *SE* = 0.05). Compared to baseline (*M* = 5.71, *SE* = 0.06), participants’ skills in navigating visits with emerging adults also improved immediately post-intervention (*M* = 5.89, *SE* = 0.03, *p* = 0.002), and this improvement was sustained over the next three months (*M* = 5.84, *SE* = 0.03, *p* = 0.07) at the *p* < 0.10 level. In addition, time had a positive linear effect on advising on symptom management at the *p* = 0.05 level, but pairwise comparisons did not show any significant differences.

For skills measured with open-ended questions, time had a positive linear effect on all skills except supporting patients, suggesting enhancement across most targeted skills. Comparing the EMMs of all skills as a group, results showed an improvement immediately post-exposure (*M* = 14.50, *SE* = 0.36) compared to baseline (*M* = 13.61, *SE* = 0.34, *p* = 0.08), although this effect did not reach conventional statistical significance. Specifically, participants showed improved skills in (1) explaining diagnosis and treatment (*M* = 2.90, *SE* = 0.10, *p* = 0.09), (2) conveying risks of antibiotics (*M* = 2.83, *SE* = 0.14, *p* = 0.048), (3) advising on symptom management (*M* = 2.40, *SE* = 0.11, *p* = 0.04), and (4) navigating visits with emerging adults (*M* = 2.34, *SE* = 0.09, *p* = 0.07) immediately post-exposure, compared to baseline (explaining diagnosis and treatment: *M* = 2.64, *SE* = 0.10; conveying risks: *M* = 2.42, *SE* = 0.14; advising on symptom management: *M* = 2.09, *SE* = 0.11; navigating emerging-adult visits: *M* = 2.13, *SE* = 0.09). It should be noted that some effects were significant at the *p* < 0.10 level. In addition, there was a long-term improvement in conveying risks of antibiotics (*M* = 2.88, *SE* = 0.13, *p* = 0.02). Overall, these findings largely supported H1. The long-term effect addressed in RQ1, however, received less support from the data.

### 2.2. Moderation Effects

All significant moderation effects are presented in Table 2. Perceived skill importance and self-efficacy in skill implementation both interacted with the linear and quadratic terms of time in predicting overall communication skills measured with close-ended questions and navigating visits with emerging adults. They also both had an interaction with the linear term of time in predicting explaining diagnosis and treatment. In addition, perceived skill importance interacted with the linear term of time in predicting supporting patients and the linear and quadratic terms of time in predicting conveying antibiotic risks. Neither variable moderated the effectiveness of the training on any skills measured with open-ended questions. Motivation to improve, on the other hand, interacted with open-ended assessments of advising on symptom management and supporting patients, along with close-ended assessments of conveying risks and navigating visits with emerging adults.

Table 3 presents the EMMs from post hoc pairwise comparisons for all significant moderation effects. Participants who perceived low (i.e., 1 *SD* below the mean) and average levels of skill importance showed an overall improvement in their communication skills that sustained over the next 14 weeks. This effect was not observed among those who perceived high importance (i.e., 1 *SD* above the mean). Participants with average and high importance perceptions showed immediate and long-term improvement in explaining diagnosis and treatment, whereas those with low importance perceptions showed only immediate improvement. For the skill of conveying risks, participants who perceived high importance experienced an immediate deterioration, a pattern not seen in other groups. Although perceived skill importance interacted with the linear term of time, pairwise comparisons showed no significant differences. Regarding the skill of navigating visits with emerging adults, participants with low and average importance perceptions exhibited both immediate and long-term improvement, while those with high importance perceptions experienced a decline from baseline to three months post-intervention.

Participants with low and average levels of self-efficacy, but not high levels, showed improvement in overall communication skills, both immediately and three months later. Those with average and high levels of self-efficacy showed immediate and long-term improvement in explaining diagnosis and treatment, while those with low self-efficacy showed only immediate improvement. Only individuals with low and average levels of self-efficacy showed immediate and long-term improvement in navigating visits with emerging adults.

No significant post hoc pairwise comparisons were found for the interaction between improvement motivation and time in predicting the skill of conveying risks. Those with low motivation showed immediate and long-term improvement in navigating visits with emerging adults, whereas those with average motivation showed only immediate improvement. Motivation also moderated the intervention effect on two skills measured with open-ended questions: Only participants with low or average motivation showed significant improvement in advising on symptom management, and only those with low motivation improved their skill in supporting patients.

## 3. Discussion

Combating antibiotic resistance is critical to preserving the effectiveness of antibiotics and our ability to treat infections. In this study, we tested the immediate and sustained effects of a series of animated intervention videos aimed at enhancing healthcare providers’ communication skills to reduce patient expectations for antibiotics while maintaining satisfaction. Overall, both open- and close-ended skill assessments supported the utility of this intervention program, particularly in the immediate term. In addition, some of the effects were moderated by individual differences. Our findings have significant implications for antibiotic stewardship and the role of health communication in this interdisciplinary effort.

The results showed significant immediate improvement in overall communication skills, assessed with both open- and close-ended measures. A closer examination of individual skill sets revealed that improvements in close-ended questions were primarily driven by enhancements in explaining diagnosis and treatment, as well as navigating visits with emerging adults. The open-ended assessment, on the other hand, showed meaningful improvements in all skills except supporting patients. The relative lack of effect in close-ended measures can likely be attributed to the small variance observed in this sample and a possible ceiling effect for some skills, such as supporting patients. Nevertheless, the results from the open-ended assessments are promising. These assessments involve translating the knowledge from the video into simulated patient–provider interactions, which requires high level cognitive processes [42,43], making them a strong indicator of the program’s utility. Supporting patients was the only open-ended skill that did not show significant improvement. The hierarchical coding scheme for this assessment, while informed by theory and research on person-/patient-centeredness [27,28], did not correspond directly with the individual skills covered in the training video, which might partially explain the lack of effect. In addition, supporting patients is likely one of the least familiar and most challenging skills to manage, as reflected by the participants’ ratings of its difficulty level. This skill thus may require a more comprehensive and in-depth intervention. Future research should continue to explore strategies that effectively help practitioners master this skill.

The long-term effectiveness of the videos was less substantiated by the findings, particularly in the open-ended assessments, where only the improvement in skills for conveying antibiotic risks was sustained over the next three months. This is perhaps not surprising given the natural decline of knowledge retention over time, which likely contributed to the observed effect of the quadratic term of time. Indeed, some past research using a similar intervention approach has also observed limited longer-term effects [37]. This suggests that instead of relying on a one-time intervention, continued efforts are necessary to improve providers’ communication skills over time. Moreover, providers may benefit more in the long term by supplementing a video-based intervention program with practice sessions that reinforce the skills learned. These sessions could help maintain high levels of competence and sustain the improvements. Additional research focusing on identifying and optimizing strategies for long-term retention could provide valuable insights into the most efficient and effective methods to prolong the gains from the intervention.

Testing of moderators revealed that the effects of the intervention videos were influenced by perceived skill importance, baseline self-efficacy, and motivation to improve. Specifically, the observed pattern suggests that overall, participants who perceived the skills as less important, had lower self-efficacy, and were less motivated to improve benefited more from the intervention compared to their counterparts with high perceived importance, efficacy, and motivation. This can be understood in light of the greater room for improvement among the former group, highlighting them as a key target audience for such interventions. The latter group, in contrast, generally exhibited higher baseline skills, which might have limited the observable improvements. Notably, those with high importance perceptions even experienced a decrease in skills for conveying risks and navigating visits with emerging adults. This decline might be due to increased pressure and anxiety stemming from the high importance they placed on these skills, negatively affecting their ability to perform them. Further, the content or delivery of the intervention may not have aligned well with the needs or preferences of this group, underscoring the necessity for future research to personalize through audience segmentation and tailored interventions. It is also worth highlighting that the open-ended assessments appeared to be less contingent on individual differences, particularly perceived skill importance and baseline self-efficacy. This indicates that the intervention was, in general, effective in enhancing participants’ ability to apply the skills in practice, regardless of their pre-existing attitudes or abilities. This finding is encouraging, as it demonstrates that the program has broad applicability and can benefit a wide range of practitioners.

The findings and implications of this study should be considered with its limitations in mind. First, although measures were implemented to prevent skipping or fast-forwarding, we could not control for participants’ engagement levels during video viewing. Future research could consider more controlled environments (e.g., in-person) to ensure consistent engagement. In addition, the animated format may not be equally engaging for all providers. Future work could diversify the format by incorporating, for example, filmed interactions featuring real providers. This would allow tailoring to learners’ preferences and help maximize effectiveness. In addition, future interventions may incorporate short-form video formats (<1 min) to deliver microlearning or booster messages. While longer videos are well-suited for demonstrating elaborated communication strategies, short-form formats may function as targeted skill primers or refreshers that capture attention efficiently, especially under time constraints in clinical settings. Second, while the intervention was specifically designed for primary healthcare providers at college health centers, the content is likely relevant to other primary care settings. However, the generalizability of these findings to different settings requires future empirical validation. Third, while the identification of the five skill sets was based on an extensive literature review and formative research, it is possible that other important skill sets were overlooked. Future research should explore additional skill sets, particularly in the context of evolving healthcare challenges, such as the COVID-19 pandemic. Fourth, we used a hypothetical scenario to assess participants’ ability to apply the skills. While this approach allowed for standardized assessment across participants, observational data from real-life patient–provider interactions would offer a more ecologically valid assessment of how these skills are enacted in practice. Future research should therefore explore integrating naturalistic interaction data (e.g., audio- or video-recorded visits), while accounting for feasibility and consent considerations. A related limitation concerns the duration of the follow-up period. Although the three-month follow-up allowed us to assess whether the intervention produced effects that persisted beyond the immediate encounter, it does not speak to longer-term maintenance. Future work should examine extended follow-up periods (e.g., 6–12 months) to determine the extent to which these effects are sustained and whether periodic reminders or booster components can support continued impact. Lastly, this study did not include a no-intervention control group, which limits our ability to rule out alternative explanations for change over time. Future research should evaluate the intervention in a controlled trial including a comparison group.

## 4. Materials and Methods

### 4.1. Material Development and Pre-Testing

The development of the intervention materials began in December 2019. Guided by existing literature and in consultation with the antibiotic stewardship committee comprising healthcare practitioners at the student health center of the last author’s institution, the research team identified the five sets of communication skills described above for the intervention. In May 2020, we launched a formative survey to understand healthcare providers’ perceptions of these five skill sets. A total of 110 providers from 50 different university health centers in the U.S. participated in the survey. Providers rated each skill as important for themselves (ranging from 4.79 to 4.82 on a 5-point scale) and their colleagues (ranging from 4.00 to 4.73). Slightly over 60% of providers rated explaining diagnosis and treatment or supporting patients as the most difficult skill to manage.

With this feedback, the research team began developing scripts for the intervention videos over the next six months. We also collaborated with an animator to develop storyboards for the videos. By the end of 2020, an initial version of the video on advising on symptom management and the storyboards for the other four videos were produced. In January 2021, we conducted a pilot test with these materials. First, we held a focus group with five healthcare providers from the student health center at the last author’s university. Providers read a program outline summarizing the objectives and components of the training program, watched the video, viewed all four storyboards, and provided feedback on the content and visuals. To gain insights from a broader group of providers, we launched a survey in February 2021, recruiting 50 providers from 27 university health centers in the U.S. to participate. All providers viewed and evaluated the video and were then randomly assigned to view and evaluate one of the four storyboards. Providers rated how appealing, understandable, credible, engaging, useful, persuasive, informative, and beneficial the video and the storyboard were on a 5-point Likert scale. Except for the engagement scale for the storyboard on explaining diagnosis and treatment (*M* = 3.52, *SD* = 1.10), ratings for the video and all storyboards on all dimensions were significantly above the scale midpoint (ranging from 3.79 to 4.74). They also responded to open-ended questions about what they liked and disliked about the content and the visuals and suggested changes. The research team closely analyzed the data and generated a list of revisions regarding the content and the visual presentations to the initial video and the storyboards. All five videos were finalized with revisions in October 2021 and are available at https://osf.io/du7ja/files?view_only=44e4719b0b0a4f6bb1863a7c4426efec (accessed on 3 December 2025). The videos were 5–7 min in length and used an animated format with professional voiceover. As previously outlined, each video provided multiple strategies corresponding to its core communication skill target: (1) explaining diagnosis and treatment: share reasoning for the diagnosis, address patient misconceptions, specify the diagnosis as viral, and explain how antibiotics relate to bacterial and viral illness; (2) conveying risks of antibiotics: find a space to talk about risks, discuss adverse effects, and explain antibiotic resistance; (3) advising on symptom management: describe symptom management as “real medicine,” emphasize effectiveness and feasibility, address advice limitations and consequences, give instructions for use, and help patients follow through; (4) supporting patients: establish a connection, draw out the patient perspective, validate patient emotions and symptoms, and emphasize the availability of follow-up care; (5) navigating visits with emerging adults: validate uncertainty, address competing sources of advice, and respect patient autonomy. For each strategy, the video explained how and why it works in practice and provided example phrasing that providers could use during visits.

### 4.2. Procedure and Participants

To test the longitudinal effectiveness of the intervention videos, we recruited providers and medical students to participate in a three-wave longitudinal study, which included a baseline survey, an immediate follow-up (2 weeks after baseline), and a final follow-up (12 weeks after the second survey). The 12-week (three-month) follow-up interval was selected because it is commonly used in intervention research to assess medium-term effects after brief training [44,45]. This duration allows for evaluation of whether skills persist beyond the immediate exposure period while maintaining participant retention and minimizing external influences that may confound longer-term effects.

The inclusion criteria, recruitment process, and study procedure are summarized in Supplementary S1. The study protocol was approved by the Institutional Review Board at the Pennsylvania State University.

Among provider participants, 108 providers took the baseline survey, 83 returned for the second survey, and 75 completed the final survey (69% retention rate). Among medical student participants, 109 took the baseline survey, 90 took the second survey, and 60 took the final survey (55% retention rate). The decline in sample size across waves reflects voluntary non-response rather than exclusion. Compared with providers who completed all three waves (*M*_age_ = 44.41), providers who dropped out after the first or second survey were older in age (*M*_age_ = 49.30, *p* = 0.02). Among medical students, dropouts and completers did not differ in gender, race, or age. In total, 135 participants completed all three waves and were included in the analyses. Provider participants in the final sample were from 55 university health centers across the United States, and medical student participants were from 45 U.S. medical schools. Their demographic information is summarized in Table 4.

### 4.3. Measures

Descriptive statistics for all variables are shown in Table 5. The close- and open-ended skill assessment items described below were developed specifically for this study because no existing measures captured the targeted stewardship communication skills. Item development was guided by the communication strategies included in the intervention and reviewed by practitioners and communication experts to ensure content validity.

*Close-ended skill assessment.* We created a 30-item closed-ended question bank to assess the communication skills covered in the five intervention videos, which is available at https://osf.io/du7ja/files/z48t2?view_only=44e4719b0b0a4f6bb1863a7c4426efec, accessed on 3 December 2025. A composite score was created by summing the number of questions answered correctly.

*Open-ended skill assessment.* Five open-ended questions were designed to assess participants’ application of the five sets of communication skills. Participants were given a hypothetical scenario and asked to record what they would say to the patient in the scenario (see Supplementary S2 for a detailed description). Phonic (https://www.phonic.ai/, accessed on 3 December 2025) was used to enable the recordings. Based on repeated readings of the data and considerations of the content and the theory underlying each video, the authors developed a coding scheme for each question and trained research assistants who were blind to the study objectives to apply the coding scheme (see Supplementary S2 for an overview of the coding scheme and procedure). The final reliability statistics are presented in Table 6. The strong inter-coder reliability indicates consistency in coding. A composite score was created by summing the coded values.

*Perceived skill importance*. In the baseline survey, participants evaluated the importance of each of the five sets of communication skills (1 = *strongly disagree*, 5 = *strongly agree*). They rated five items corresponding to each skill set (e.g., “It is important for me to be skilled at explaining why diagnoses do not warrant antibiotic treatment”).

*Perceived self-efficacy in implementing the skills*. In the baseline survey, participants reported their self-efficacy in implementing each skill (1 = *strongly disagree*, 5 = *strongly agree*) using five items (e.g., “I am confident in my ability to explain why diagnoses do not warrant antibiotic treatment”).

*Skill improvement motivation*. After watching the videos in the second survey, participants indicated their motivation to improve each of the five skill sets described in the videos. For each skill set, participants reported their motivation to improve the specific skills described in the video (1 = *no motivation at all*, 5 = *a great deal of motivation*). A composite score indexing improvement motivation for each skill set was created by averaging the ratings across the corresponding items. An overall score indicating improvement motivation for all skills was also created by averaging the ratings across all items.

### 4.4. Data Analyses

To test the hypothesis and address the research questions, mixed-effects models were estimated using the nlme package (Version 3.1-166) [46] in R (Version 4.5.0) [47]. This approach is suitable for handling the nested structure of repeated measures within participants. With measurement taken at baseline, two weeks post-baseline (immediate post-intervention), and fourteen weeks post-baseline (three months post-intervention), time was treated as a continuous variable, coded as 0, 2, and 14 for the three points, respectively, to reflect the actual time intervals. For models testing H1 and addressing RQ1, the fixed effects included an intercept, a linear term for time, and a quadratic term for time. The inclusion of the quadratic term was based on preliminary analyses indicating potential non-linear trends in skill performance over time. Following the model building approach described in Shiverdecker and Lebreton [48], we estimated a series of models with random intercepts only, random intercepts and random slopes for the linear term of time, and random intercepts and random slopes for both the linear and quadratic terms of time. Model comparisons were conducted to determine the most parsimonious model. For example, the quadratic term of time was not included in the random effects structure when model comparisons showed that it did not improve model fit compared to the model with only random slopes for the linear term of time. Post hoc pairwise comparisons of estimated marginal means were conducted using the emmeans package (Version 1.8.7) [49]. To address RQ2, the interaction term between the linear term of time and each moderator, centered at the sample-level mean and tested one at a time, was first added to the models. The models were then expanded to include the interaction term between the quadratic term of time and each moderator. We also explored whether the effects varied between providers and medical student participants. Participant type did not significantly moderate any of the effects. Therefore, the results reported below are based on the combined sample.

## 5. Conclusions

This study provides evidence supporting the effectiveness of a short, animated video-based intervention for improving providers’ antibiotic stewardship communication skills. We observed immediate post-intervention improvement in most skills, although our test of long-term impact underscores the need for ongoing reinforcement to sustain these gains. Notably, the intervention was particularly beneficial for individuals with initially low motivation and self-efficacy, a crucial target group for such programs. Overall, this study sheds light on the potential of cost-efficient, communication-based interventions in enhancing antibiotic stewardship and demonstrates the vital role health communication plays in addressing the global health crisis of antibiotic resistance.

## Figures and Tables

**Table 1:** Fixed Effects Predicting Communication Skill Assessments.

	Intercept			Time			Time^2^		
	Coef.	*SE*	*p*	Coef.	*SE*	*p*	Coef.	*SE*	*p*
**Close-ended Skill Assessments (all)**	28.25	0.12	<0.001	0.27	0.08	<0.001	−0.02	0.01	0.002
Explaining diagnosis and treatment	5.60	0.05	<0.001	0.14	0.03	<0.001	−0.01	0.00	<0.001
Conveying antibiotic risks	5.79	0.04	<0.001	−0.01	0.03	0.66	0.00	0.00	0.65
Advising on symptom management	5.21	0.05	<0.001	0.07	0.04	0.05	−0.00	0.00	0.06
Supporting patients	5.94	0.02	<0.001	−0.03	0.02	0.18	0.00	0.00	0.21
Navigating visits w/emerging adults	5.71	0.06	<0.001	0.10	0.03	<0.001	−0.01	0.00	<0.001
**Open-ended Skill Assessments (all)**	13.61	0.34	<0.001	0.52	0.24	0.03	−0.03	0.02	0.04
Explaining diagnosis and treatment	2.64	0.10	<0.001	0.15	0.07	0.04	−0.01	0.00	0.04
Conveying antibiotic risks	2.42	0.14	<0.001	0.23	0.10	0.02	−0.01	0.01	0.03
Advising on symptom management	2.09	0.11	<0.001	0.18	0.08	0.02	−0.01	0.01	0.01
Supporting patients	4.00	0.14	<0.001	0.03	0.11	0.77	−0.00	0.01	0.78
Navigating visits w/emerging adults	2.13	0.09	<0.001	0.12	0.05	0.03	−0.01	0.00	0.04

Note. There were no missing data for the close-ended assessments. Missing data for the open-ended assessments were handled with casewise deletion.

**Table 2:** Moderation Effects.

	Time × Importance		Time^2^ × Importance		Time × Efficacy		Time^2^ × Efficacy		Time × Motivation		Time^2^ × Motivation	
	Coef.	*SE*	Coef.	*SE*	Coef.	*SE*	Coef.	*SE*	Coef.	*SE*	Coef.	*SE*
**All_C**	−0.76	0.21	0.05	0.02	−0.43	0.17	0.03	0.01	--	--	--	--
S1	0.04	0.01	--	--	0.01	0.01	--	--	--	--	--	--
S2	−0.35	0.12	0.02	0.01	--	--	--	--	0.10	0.04	−0.01	0.00
S3	--	--	--	--	--	--	--	--	--	--	--	--
S4	−0.01	0.01	--	--	--	--	--	--	--	--	--	--
S5	−0.57	0.10	0.03	0.01	−0.15	0.07	0.01	0.00	−0.13	0.04	0.01	0.00
**All_O**	--	--	--	--	--	--	--	--	--	--	--	--
S1	--	--	--	--	--	--	--	--	--	--	--	--
S2	--	--	--	--	--	--	--	--	--	--	--	--
S3	--	--	--	--	--	--	--	--	−0.23	0.10	0.01	0.01
S4	--	--	--	--	--	--	--	--	−0.49	0.15	0.03	0.01
S5	--	--	--	--	--	--	--	--	--	--	--	--

Note. All_C = All communication skills measured with close-ended questions; All_O = All communication skills measured with open-ended questions. S1 = explaining diagnosis and treatment; S2 = conveying antibiotic risks; S3 = advising on symptom management; S4 = supporting patients; S5 = navigating visits with emerging adults. Only effects significant at the *p* < 0.05 level are shown.

**Table 3:** Estimated Marginal Means at Different Levels of Moderators.

Moderator	Level	Week 0	Week 2	Week 14
Importance		Close-Ended Skill Assessments (All)		
	−1 SD	27.52 (0.15) ^a^	28.31 (0.16) ^b^	28.25 (0.13) ^b^
	Mean	28.25 (0.11) ^a^	28.72 (0.11) ^b^	28.67 (0.09) ^b^
	+1 SD	28.99 (0.15)	29.12 (0.16)	29.08 (0.13)
		Close-Ended Explaining Diagnosis and Treatment		
	−1 SD	5.58 (0.06) ^a^	5.80 (0.04) ^b^	5.66 (0.04) ^a^
	Mean	5.60 (0.05) ^a^	5.84 (0.03) ^b^	5.84 (0.03) ^b^
	+1 SD	5.62 (0.06) ^a^	5.89 (0.04) ^b^	6.01 (0.04) ^b^
		Close-Ended Conveying Risks		
	−1 SD	5.57 (0.06)	5.70 (0.06)	5.70 (0.06)
	Mean	5.79 (0.04)	5.77 (0.04)	5.79 (0.04)
	+1 SD	6.02 (0.06) ^a^	5.84 (0.06) ^b^	5.89 (0.06)
		Close-Ended Supporting Patients		
	−1 SD	5.85 (0.03)	5.80 (0.04)	5.87 (0.04)
	Mean	5.94 (0.02)	5.89 (0.03)	5.91 (0.03)
	+1 SD	6.03 (0.03)	5.98 (0.04)	5.95 (0.04)
		Close-Ended Navigating Visits with Emerging Adults		
	−1 SD	5.29 (0.05) ^a^	5.72 (0.05) ^b^	5.74 (0.05) ^b^
	Mean	5.71 (0.04) ^a^	5.89 (0.04) ^b^	5.84 (0.03) ^b^
	+1 SD	6.13 (0.05) ^a^	6.06 (0.05)	5.95 (0.05) ^b^
Efficacy		Close-Ended Skill Assessments (All)		
	−1 SD	27.98 (0.17) ^a^	28.78 (0.16) ^b^	28.54 (0.14) ^b^
	Mean	28.25 (0.12) ^a^	28.72 (0.12) ^b^	28.67 (0.10) ^b^
	+1 SD	28.52 (0.17)	28.65 (0.16)	28.79 (0.14)
		Close-Ended Explaining Diagnosis and Treatment		
	−1 SD	5.60 (0.06) ^a^	5.84 (0.04) ^b^	5.76 (0.04)
	Mean	5.60 (0.05) ^a^	5.84 (0.03) ^b^	5.84 (0.03) ^b^
	+1 SD	5.60 (0.06) ^a^	5.85 (0.04) ^b^	5.92 (0.04) ^b^
		Close-Ended Navigating Visits with Emerging Adults		
	−1 SD	5.55 (0.08) ^a^	5.84 (0.04) ^b^	5.81 (0.04) ^b^
	Mean	5.71 (0.06) ^a^	5.89 (0.03) ^b^	5.84 (0.03) ^b^
	+1 SD	5.87 (0.08)	5.94 (0.04)	5.88 (0.04)
Motivation		Close-Ended Conveying Risks		
	−1 SD	5.83 (0.06)	5.69 (0.06)	5.82 (0.06)
	Mean	5.79 (0.04)	5.77 (0.04)	5.79 (0.04)
	+1 SD	5.76 (0.06)	5.85 (0.06)	5.77 (0.06)
		Close-Ended Navigating Visits with Emerging Adults		
	−1 SD	5.53 (0.08) ^a^	5.87 (0.04) ^b^	5.77 (0.05) ^b^
	Mean	5.71 (0.06) ^a^	5.89 (0.03) ^b^	5.84 (0.03)
	+1 SD	5.90 (0.08)	5.90 (0.04)	5.92 (0.05)
		Open-Ended Advising on Symptom Management		
	−1 SD	1.78 (0.16) ^a^	2.38 (0.16) ^b^	2.07 (0.17)
	Mean	2.08 (0.11) ^a^	2.41 (0.11) ^b^	2.17 (0.11)
	+1 SD	2.38 (0.15)	2.43 (0.16)	2.26 (0.16)
		Open-Ended Supporting Patients		
	−1 SD	3.49 (0.21) ^a^	4.23 (0.22) ^b^	4.00 (0.20)
	Mean	3.97 (0.14)	4.06 (0.15)	4.04 (0.14)
	+1 SD	4.46 (0.20)	3.90 (0.21)	4.09 (0.20)

Note. SEs are shown in parentheses. Values with different superscripts in the same row are different at *p* < 0.05.

**Table 4:** Sample Demographics.

	Providers		Medical Students	
Demographic Characteristics	*n*	Percentage	*n*	Percentage
**Gender**				
Female	64	85.33	41	68.33
Male	11	14.67	19	31.67
**Race**				
White	67	89.33	23	38.33
Black or African American	5	6.67	3	5.00
Asian	2	2.67	27	45.00
American Indian or Alaska Native	--	--	1	1.67
More than one race	--	--	3	5.00
Other or prefer not to answer	1	1.33	3	5.00
**Ethnicity**				
Hispanic or Latino	2	2.67	2	3.33
Not Hispanic or Latino	73	97.33	58	96.67
**Medical Qualification**				
Doctor of Osteopathic Medicine	2	2.67	--	--
Doctor of Medicine	11	14.67	--	--
Nurse Practitioner	48	64.00	--	--
Physician Assistant	14	18.67	--	--
Third-year medical student	--	--	25	41.67
Fourth-year medical student	--	--	35	58.33
	***M***	***SD***	***M***	***SD***
**Age**	44.41	8.82	26.81	2.41
**Total year practicing medicine**	14.86	8.47	--	--
**Year practicing medicine at a student health center**	7.35	5.93	--	--
**Hours work per week**	36.26	10.36	--	--
**Patients see per week**	49.16	27.56	--	--
**URI patients see per week during cold/flu season**	47.97	65.25	--	--

Note. *n* = number of participants in each subgroup. *M* = mean. *SD* = standard deviation. URI = upper respiratory tract infection. Providers were from 55 college or university student health centers. Medical students were from 45 medical schools.

**Table 5:** Descriptive Statistics.

	T1			T2			T3	
	*M*	*SD*	α	*M*	*SD*	α	*M*	*SD*
**Close-Ended Skill Assessment**	28.25	1.43	--	28.72	1.35	--	28.67	1.13
Explaining diagnosis and treatment	5.60	0.58	--	5.84	0.36	--	5.84	0.39
Conveying antibiotic risks	5.79	0.46	--	5.77	0.53	--	5.79	0.46
Advising on symptom management	5.21	0.67	--	5.33	0.61	--	5.28	0.59
Supporting patients	5.94	0.24	--	5.89	0.40	--	5.91	0.31
Navigating visits w/emerging adults	5.71	0.68	--	5.89	0.34	--	5.84	0.40
**Open-Ended Skill Assessment**	13.60	3.05	--	14.60	3.32	--	14.10	3.84
Explaining diagnosis and treatment	2.65	0.99	--	2.93	0.96	--	2.75	1.13
Conveying antibiotic risks	2.41	1.30	--	2.86	1.46	--	2.90	1.54
Advising on symptom management	2.10	1.12	--	2.40	1.29	--	2.15	1.17
Supporting patients	3.99	1.60	--	4.06	1.43	--	4.03	1.55
Navigating visits w/emerging adults	2.15	0.94	--	2.36	0.94	--	2.31	0.92
**Importance**	4.93	0.25	0.85	--	--	--	--	--
Explaining diagnosis and treatment	4.93	0.34	--	--	--	--	--	--
Conveying antibiotic risks	4.92	0.35	--	--	--	--	--	--
Advising on symptom management	4.95	0.22	--	--	--	--	--	--
Supporting patients	4.96	0.30	--	--	--	--	--	--
Navigating visits w/emerging adults	4.92	0.37	--	--	--	--	--	--
**Self-Efficacy**	4.54	0.45	0.79	--	--	--	--	--
Explaining diagnosis and treatment	4.56	0.61	--	--	--	--	--	--
Conveying antibiotic risks	4.36	0.71	--	--	--	--	--	--
Advising on symptom management	4.61	0.61	--	--	--	--	--	--
Supporting patients	4.62	0.54	--	--	--	--	--	--
Navigating visits w/emerging adults	4.53	0.61	--	--	--	--	--	--
**Motivation**	--	--	--	4.32	0.66	0.97	--	--
Explaining diagnosis and treatment	--	--	--	4.33	0.74	0.94	--	--
Conveying antibiotic risks	--	--	--	4.26	0.70	0.85	--	--
Advising on symptom management	--	--	--	4.27	0.71	0.89	--	--
Supporting patients	--	--	--	4.37	0.76	0.94	--	--
Navigating visits w/emerging adults	--	--	--	4.38	0.73	0.91	--	--

Note. T1 = baseline (pre-intervention); T2 = immediate post-intervention; T3 = three-month follow-up. *M* = mean. *SD* = standard deviation.

**Table 6:** Coding Reliability.

	Krippendorff’s α
**Explaining diagnosis and treatment** (*N* = 4)	
Share reasoning for diagnosis	0.78
Address patient misconceptions	1.00
Specify diagnosis as viral	1.00
Explain how antibiotics relate to bacterial and viral illness	0.96
Full explanation	0.77
**Conveying antibiotic risks** (*N* = 4)	
Discuss adverse effects of antibiotics	0.95
Explain antibiotic resistance	0.81
Describe resistance in a way that includes bacterial adaptation	0.81
Discuss antibiotic resistance severity and susceptibility	0.74
Connect unnecessary use of antibiotics to antibiotic resistance	0.82
**Advising on symptom management** (*N* = 2)	
Effectiveness	0.78
Positive feasibility	0.76
Negative feasibility	0.93
Limitations	1.00
Consequences	1.00
Give instructions	0.72
Help patient follow-through	0.87
**Supporting patients** (*N* = 3)	
Level of support quality	0.76
**Navigating visits with emerging adults** (*N* = 5)	
Acknowledge emerging adulthood	0.97
Foster the relationship	0.90
Gather information	0.90
Enable treatment-related behavior	0.90

Note. *N* indicates the number of coders. “Full explanation” is a follow-up category of “explain how antibiotics relate to bacterial and viral illness,” capturing whether the explanation fully and explicitly states that antibiotics only work for bacterial infections, not viral infections.

## Data Availability

The data underlying this article will be shared on reasonable request to the corresponding author.

## Supplementary Materials

The following supporting information can be downloaded at: https://www.mdpi.com/article/10.3390/antibiotics14121270/s1. Supplementary S1. Additional Details on Procedure and Participants. Supplementary S2. Open-Ended Assessment.
