# Supplementary material for: Leveraging Communication to Combat Antibiotic Resistance: A Longitudinal Test of a Video-Based Intervention to Improve Providers’ Stewardship Skills
Source: Antibiotics (Basel). 2025 Dec 15;14(12):1270. doi: 10.3390/antibiotics14121270 (PMC12730130; doi:10.3390/antibiotics14121270)
Supplement: Supplementary file 1 [file antibiotics-14-01270-s001.zip › antibiotics-4024680-supplementary.pdf]

## Supplementary Materials

### Supplementary S1. Additional Details on Procedure and Participants

Providers had to meet the following criteria to be eligible: (1) be a Doctor of Medicine, Doctor of Osteopathic Medicine, nurse practitioner, or physician assistant, and (2) be affiliated with and actively practicing medicine at a university or college student health center in the U.S. at the time of data collection. Medical students had to be (1) attending a medical school in the U.S. and (2) in their third or fourth year to be eligible.

To recruit providers, the research team emailed medical directors of student health centers at four-year, large and medium-sized, highly and primarily residential colleges per the Carnegie Classification of Institutions of Higher Education, inviting them to forward the study information to their providers. Medical students were recruited through either recruitment emails sent to directors at medical schools or a recruitment advertisement published with the American Medical Student Association.

Consented and eligible participants first completed a baseline survey where they reported their demographics and perceived importance of and self-efficacy in practicing the five sets of communication skills. They also responded to close- and open-ended questions assessing their communication skills corresponding to those covered in the five videos. Two weeks after completing the first survey, participants were sent a link to the second survey. Participants watched all five videos embedded in this survey. Each video was played once. After that, participants again responded to questions assessing the five sets of skills and reported their motivations to improve each skill. Finally, 12 weeks (3 months) after completing the second survey, participants were sent the third and final survey, which included the same skill assessment questions as those in the first and second surveys. Data collection occurred from November 2021 to September 2022.

**Supplementary S2. Open-Ended Assessment**
**<u>Hypothetical Scenario and Question Prompts</u>**
The following section contains five open-ended, audio-based questions. For each of the following questions, please imagine the following scenario:

You are seeing Sam, a college student (age 20) with an upper respiratory tract infection. You take Sam's medical history, discuss current symptoms, and complete the physical exam. Sam expresses discomfort with the symptoms, which include a cough, congestion, headache, and fatigue. Sam also mentions stress related to being ill and unable to do normal activities. Testing indicates that Sam does not have COVID-19, and testing for flu or strep throat doesn't appear warranted. You believe that Sam has a viral infection that does not require antibiotics. Instead, you intend to recommend non-antibiotic treatment options such as over-the-counter medications.

1. *How would you explain your diagnosis and treatment recommendation to Sam? Please imagine that you are currently taking to Sam, start recording, and speak what you would say to Sam. If you would not say anything, say "I wouldn't talk about this."*
2. *How would you discuss the risks of antibiotics with Sam? Please imagine that you are currently taking to Sam, start recording, and speak what you would say to Sam. If you would not say anything, say "I wouldn't talk about this."*
3. *How would you advise Sam on symptom management? Please imagine that you are currently taking to Sam, start recording, and speak what you would say to Sam. If you would not say anything, say "I wouldn't talk about this."*
4. *How would you try to support Sam (i.e., respond to Sam's expressions of discomfort and stress)? Please imagine that you are currently taking to Sam, start recording, and speak what you would say to Sam. If you would not say anything, say "I wouldn't talk about this."*
5. *How would your overall approach to talking with Sam take into account their status as a young adult and college student? Please be as specific as possible. If your approach would not take this into account, say "I wouldn't take this into account."*

**<u>Coding Scheme</u>**
*Skill 1: Explaining Diagnosis and Treatment* (0 = absent, 1 = present)
a. Share reasoning for the diagnosis
b. Address patient misconceptions
c. Specify the diagnosis as viral
d. Explain how antibiotics relate to bacterial and viral illness
    i. Does the above explanation fully explain that antibiotics only work for bacterial infections, not viral infections?

*Skill 2: Conveying the Risks of Antibiotics* (0 = absent, 1 = present)
a. Discuss the adverse effects of antibiotics
b. Explain antibiotic resistance
c. Describe resistance in a way that includes bacterial adaptation
d. Discuss the severity of the threat from antibiotic resistance and susceptibility to it
e. Connect unnecessary use of antibiotics to antibiotic resistance

*Skill 3: Advising on Symptom Management* (0 = absent, 1 = present)

a. Discuss effectiveness
b. Discuss positive feasibility
c. Discuss negative feasibility
d. Discuss limitations
e. Discuss consequences
f. Give instructions for use
g. Help patients follow through

*Skill 4: Supporting Patients* (1-5)
a. Level of support quality
    1. Give advice
    2. Offer assistance
    3. Express sympathy
    4. Provide situational explanation
    5. Validate emotions

*Skill 5: Navigating Medical Visits with Emerging Adults* (0 = absent, 1 = present)
a. Acknowledge emerging adulthood
b. Foster the relationship
c. Gather information
d. Enable treatment-related behavior

**Coding Procedure**

The following process was followed to code the data for each question: (1) The authors hold an initial meeting with the research assistants (RAs) to familiarize them with the coding scheme and answer questions. (2) Approximately 15 responses were randomly drawn from the data as a practice sample. The authors and RAs all code the sample. (3) A second meeting is held to compare the coding and resolve any disagreements. In a few cases where disagreements are high, the team codes an additional practice sample for discussion. (4) Upon completing the practice sample(s), RAs are given the first reliability sample (8-10% responses randomly drawn from the data) to code. Regardless of the reliability results, a third meeting is held to resolve any disagreements. If reliability is achieved, the remaining data are divided, and each RA independently codes their assigned data. If not, the process repeats with new samples until ideal reliability is achieved.
